# Complementary Strategies for the Management of Radiation Therapy Side Effects

**DOI:** 10.6004/jadpro.2013.4.4.3

**Published:** 2013-07-01

**Authors:** Christine E. Stubbe, Meighan Valero

**Affiliations:** Dr. Stubbe recently completed a 3-year residency at the Yellowstone Naturopathic Clinic and Frontier Cancer Center, Billings, Montana. Dr. Valero is a naturopathic doctor and researcher currently practicing in Windsor, Ontario.

## Abstract

Patients with cancer utilize complementary and alternative medicine (CAM) for a variety of purposes, one of which is the reduction of side effects of conventional treatment. With a large number of their patients using CAM, it is important for advanced practitioners in oncology to have an understanding of these therapies to better guide their patients. Side effects of radiation therapy that may have dose-limiting poten­tial include diarrhea, mucositis, skin toxicity, and xerostomia. A com­mon side effect that is not necessarily dose-limiting but considerably troublesome to patients is cancer- and treatment-related fatigue. The CAM therapies that may alleviate some of the side effects of radiation therapy include probiotics, psyllium, exercise, melatonin, honey, acu­puncture, and calendula. Therapies that require more research or have been shown to be ineffective include aloe vera, glutamine, and deglyc­yrrhizinated licorice. This article provides an overview of these thera­pies as well as related research and analysis.

It is well established that complementary and alternative medicine (CAM) is widely used by patients with cancer for a variety of reasons (Ge et al., 2012; Gilett, Ientile, Hiscock, Plank, & Martin, 2012; Moran et al., 2012). Patients may use CAM because of a clear desire to improve their quality of life, control or cure their disease, prevent a recurrence of cancer, relieve cancer-related symptoms, or reduce the side effects of conventional treatment.

Recent studies have focused on CAM usage among patients undergoing radiation therapy (RT) for cancer treatment. One study showed that 38% of patients receiving chemoradiation therapy used CAM at one point or another (Gilett et al., 2012). Only 40% of these patients discussed CAM usage with their health-care provider. Another multicenter study of patients with breast cancer undergoing radiation therapy reported 54% as having used CAM (Moran et al., 2012). Of this group, only 16% were advised by a medical professional prior to beginning the CAM therapy. Another study of patients undergoing RT showed 95% CAM use; 47% of those did not disclose use of CAM to their providers (Rausch et al., 2011). With such a large number of patients using CAM therapy, it is important for the advanced practitioner (AP) in oncology to be aware of current common CAM practices.

The umbrella term CAM encompasses a wide range of therapeutic modalities, including—among other interventions—herbal products and nutritional supplements that have the potential to interact with standard treatments for cancer. But CAM also encompasses other modalities such as exercise and acupuncture that are not supplements or nutritional substances. Research in radiation and CAM primarily focuses on the side-effect–mitigating properties of certain nutrients as well as the enhancement of RT efficacy. It is beneficial for the oncology team to be aware of safe and effective CAM strategies that their patients can potentially integrate during the course of radiation therapy.

he primary focus of this article is to give the AP tools to help decrease the treatment-related side effects that may have dose-limiting potential. We put an emphasis on simple, cost-effective strategies that patients can implement relatively easily. Hyperbaric oxygen therapy for chronic symptoms resulting from radiation to the pelvis—proctitis, cystitis, soft-tissue necrosis, and osteonecrosis (Craighead et al., 2011)—and laser therapy for the treatment of mucositis (Worthington et al., 2011) are indeed supported in the literature, but they may not be realistic options for patients as resources may be limited or the equipment and training may not be available. The contraindications of selected therapies are also discussed.

The benefits of discussing a healthy diet with patients should not be overlooked, but diet and nutrition will not be covered thoroughly in this review. A randomized controlled study found that dietary counseling significantly improved outcomes in patients undergoing radiation therapy for colon cancer compared to patients who either had no counseling or who were given a high-protein supplemental beverage (Rock, 2005). Nutritional status and quality of life improved, with a decrease in morbidity, in the group who received nutritional counseling. A comprehensive approach to managing patients undergoing RT would ideally involve therapeutic nutritional counseling and advice on any supplementation or other modalities available to ameliorate toxicities and/or enhance efficacy of treatment.

## Diarrhea

Diarrhea occurs at a rate of up to 50% in patients receiving radiation to the pelvis or abdomen; incidence is higher with concurrent chemotherapy (Muehlbauer et al., 2009). Consequences of diarrhea include dehydration, electrolyte imbalance, malnutrition, and hospitalization, and it may have a dose-limiting effect.

**GLUTAMINE**

Numerous trials support the concurrent use of glutamine with certain chemotherapy regimens in the prevention and treatment of chemotherapy-induced diarrhea as well as the treatment of other chemotherapy side effects. Glutamine improves GI repair, as it is the preferred fuel for enterocytes. The results of larger trials using glutamine to prevent enteritis in patients undergoing radiation are not as promising as those seen in a few small trials (Membrive Conejo et al., 2011; Rotovnik Kozjek et al., 2011; Kozelsky et al., 2003). Glutamine may be considered for use in concurrent chemoradiation regimens, as the ameliorative effect on diarrhea and mucositis with certain chemotherapy drugs is beneficial, especially when dose limitation is considered.

**PROBIOTICS**

Probiotics are beneficial microorganisms that populate the GI tract and maintain balance between pro- and anti-inflammatory cytokines (Visich & Yeo, 2010). They modulate immune activity and epithelial function in the large and small intestines. Probiotics have been studied extensively and have been shown to be beneficial in patients with inflammatory bowel disease and irritable bowel syndrome. The disturbance in the gut flora is one of the mechanisms underlying the pathophysiology of radiation enteritis or colitis (Delia et al., 2007).

A systematic review and meta-analysis performed by Fuccio et al. (2009) concluded that probiotics are beneficial in the prevention and treatment of radiation-induced diarrhea in animal studies. There were encouraging results in human studies, though the few studies included in the review did not provide firm conclusions. A double-blind, placebo-controlled trial performed on 490 patients receiving adjuvant postoperative radiation therapy for cervical, sigmoid, or rectal cancer showed that probiotic administration provided protection against radiation-induced diarrhea (Delia et al., 2007). The commercial broad-spectrum probiotic preparation VSL#3 was administered as one sachet three times daily compared with the control group, who received a placebo sachet. Supplementation began on the first day of radiation treatment and continued daily through the last day of radiation treatment. Radiation-induced enteritis and colitis were observed more in the placebo group compared to the probiotic group (51.8% vs. 31.6%; * p* < .001). Patients in the placebo group suffered more grade 3/4 diarrhea (World Health Organization [WHO] grading scale) than the probiotic group (55.4% vs. 1.4%, * p* < .001).

A randomized, double-blind, placebo-controlled study of 63 patients with cervical cancer undergoing concurrent pelvic radiotherapy and weekly cisplatin showed benefit with the use of a probiotic (Infloran, see below for active ingredients) against radiation-induced diarrhea (Chitapanarux et al., 2010). All patients took two capsules of either probiotics or placebo twice daily beginning 7 days prior to the start of treatment and continuing daily through the end of treatment. The rates of grades 1, 2, and 3 diarrhea (using National Cancer Institute Common Toxicity Criteria) were 55%, 42%, and 3%, respectively, in the placebo group, and 91%, 9%, and 0% in the probiotic group.

According to Chitapanarux et al. (2010) and Delia et al. (2007), the particular strains of bacteria, the combination of more than one strain, and the concentration all contributed to the success of these studies. VSL#3 contains 450 billion/g of viable lyophilized bacteria, including four strains of lactobacilli (Lactobacillus casei, Lactobacillus plantarum, Lactobacillus acidophilus, and Lactobacillus delbrueckii, subspecies bulgaricus), three strains of bifidobacteria (Bifidobacterium longum, Bifidobacterium breve, and Bifidobacterium infantis), and one strain of Streptococcus salivarius, subspecies thermophilus. Infloran contains 2 million L. acidophilus and Bifidobacterium bifidum per capsule.

A systematic review conducted by the Mucositis Study Group of the Multinational Association of Supportive Care in Cancer/International Society of Oral Oncology (MASCC/ISOO) produced the most updated guideline regarding probiotic treatment of GI mucositis in patients with cancer. The newest guideline "…suggests that probiotic treatment containing Lactobacillus species may be beneficial for prevention of chemotherapy- and radiotherapy-induced diarrhea…" (Gibson et al., 2013, p. 315). The review could not recommend a dosage/regimen given that the studies to date investigated a wide variety of products.

Although bacteremia was not observed in any human studies on probiotic administration during radiation therapy, caution must be exercised in cases of neutropenia, where supplementation of live bacteria may become infective. There are case reports of L. acidophilus bacteremia in the literature (Ledoux, Labombardi, & Karter, 2006). Neutropenia is not a concern in patients who are receiving radiation that is not expected to cause myelosuppression. Patients receiving concurrent myelosuppressive chemotherapy regimens, radiation to the marrow-containing bones, or other immunosuppressive therapies should be monitored more closely with probiotic usage.

**PSYLLIUM**

Psyllium seed husk is a soluble fiber that is used for the treatment of several GI diseases, including constipation, diarrhea, inflammatory bowel disease, and irritable bowel syndrome (Singh, 2007). A review by Muehlbauer et al. (2009) for a Putting Evidence Into Practice guideline concluded that soluble fiber is likely to be effective for treating chemotherapy- or radiotherapy-induced diarrhea and that additional research determining the type of fiber and dose is needed. In a pilot study in which 60 patients receiving pelvic radiation were randomized to receive a psyllium bulking agent (n = 30) or not, there was a statistically significant reduction in the incidence *(p* =.049) and severity *(p* =.030) of diarrhea in the psyllium group, using 1 to 2 tsp daily (Murphy, Stacey, Crook, Thompson, & Panetta, 2000).

The authors recommend psyllium husk powder that does not contain additives. Slow introduction of fiber can minimize abdominal distension, gas, and bloating. Given that psyllium delays gastric emptying and reduces the acceleration of colon transit (Singh, 2007), caution to prevent intestinal blockage is advised in conditions of decreased gut motility and in patients taking opioid pain medications. Individuals who have been advised to follow a low-residue diet should avoid psyllium fiber.

## Fatigue

Fatigue is a common symptom experienced by patients receiving RT, though it is not usually so severe that it is dose-limiting. An evaluation of fatigue is multifaceted and may involve multiple contributing factors (Mustian et al., 2007).

**EXERCISE**

A growing body of research shows that physical activity alleviates acute cancer-related fatigue both during and after completion of RT (Mustian et al., 2007). The National Comprehensive Cancer Network (NCCN) guidelines recommend endurance and resistance exercise during and after treatment for fatigue (NCCN, 2013). Results of studies show that exercise is safe and well tolerated by patients with various cancer diagnoses receiving a range of conventional treatments. A systematic review conducted by Kuchinski, Reading, and Lash (2009) supported the inclusion of scheduled exercise to ameliorate fatigue in patients during cancer treatment. Professional support to facilitate patient compliance was encouraged. See Table 1 for a comprehensive list of examples of exercise methods studied in the systematic review by Kuchinski and colleagues.

**Table 1:**
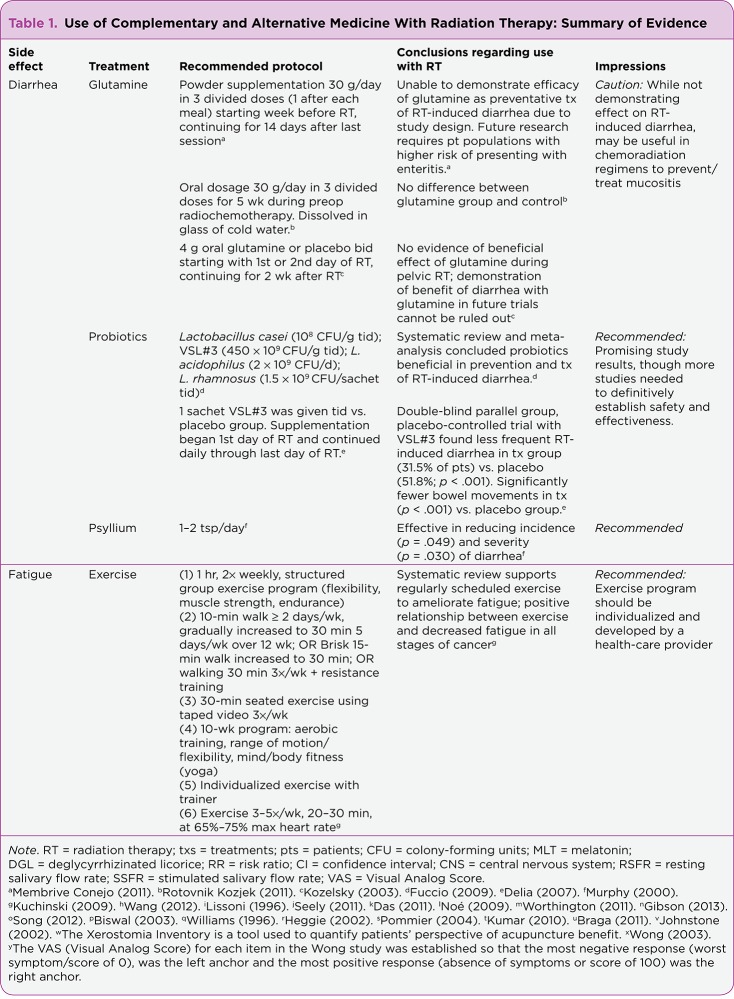
Table 1.Use of Complementary and Alternative Medicine With Radiation Therapy: Summary of Evidence

**Table 1:**
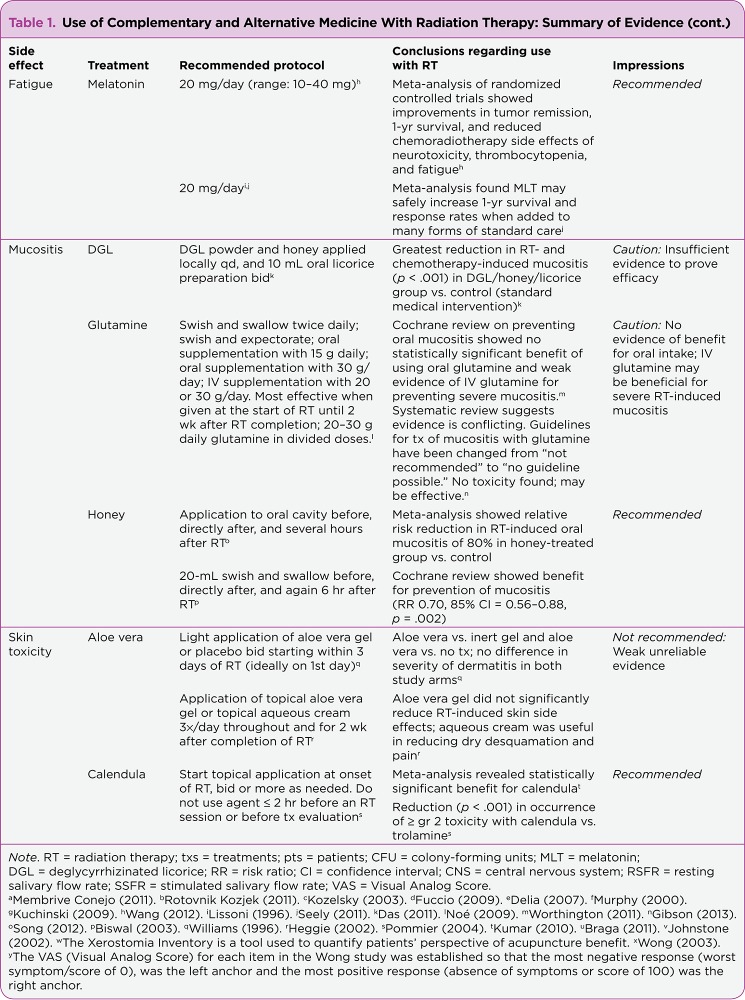
Table 1.Use of Complementary and Alternative Medicine With Radiation Therapy: Summary of Evidence (cont.)

**Table 1:**
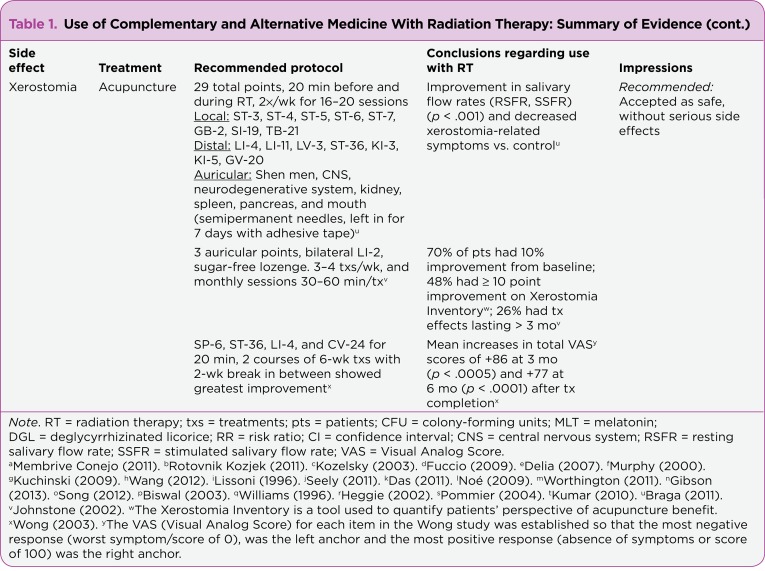
Table 1.Use of Complementary and Alternative Medicine With Radiation Therapy: Summary of Evidence (cont.)

**MELATONIN**

Melatonin (MLT), a hormone produced by the pineal gland that is released in response to a darkened environment, plays an important role in circadian rhythms (Sanchez-Barcelo, Mediavilla, Alonso-Gonzalez, & Reiter, 2012). Melatonin is helpful for the treatment of insomnia, which may lead to improved quality of sleep and less fatigue. A recent meta-analysis of randomized, controlled trials on MLT showed consistent improvements in tumor remission and 1-year survival, and a reduction in the chemoradiotherapy side effects of neurotoxicity, thrombocytopenia, and fatigue (Wang et al., 2012). Another meta-analysis of MLT by Seely et al. (2011) reached similar conclusions. Both of these meta-analyses only included one study on MLT and RT, with the remaining studies pertaining to supplementation of MLT with chemotherapy.

The singular study on MLT with RT involved 30 patients with glioblastoma receiving RT. The group receiving MLT with RT had a statistically significant benefit (* p* < .02) of increased survival at 1 year and fewer RT- or steroid-related toxicities (Lissoni et al., 1996). The researchers observed improved quality of life in the MLT-treated group with relief of anxiety and improvement in sleep and dreams. The dose used in most studies included in these meta-analyses was 20 mg/day, with a range of 10 to 40 mg.

The benefits of MLT for patients with breast cancer further extend to include selective estrogen receptor modulator (SERM) and selective estrogen enzyme modulator (SEEM) properties. SERMs selectively stimulate or inhibit the estrogen receptors of different target tissues, preventing the activation of genes that stimulate cell proliferation and therefore preventing the growth of breast cancer cells. Melatonin also has SEEM properties, which act to decrease the biotransformation of estrogens from adrenal androgens. Melatonin therefore has an impact on osteoporosis and hormonal risk factors in addition to ameliorating fatigue from radiation therapy (Sanchez-Barcelo et al., 2012; Seely et al., 2011). Epidemiologic and experimental evidence correlate disruption of nocturnal MLT secretion with increased risk of breast cancer. This effect can be observed in night shift workers (Richter et al., 2001).

Based on the authors’ clinical experience, it is wise to advise increasing the melatonin dose slowly, as side effects of morning drowsiness, vivid dreams, and mild headache may be experienced. Due to its immunostimulatory properties (Seely et al., 2011; Wang et al., 2012), caution is recommended with MLT use and cancers of the immune system. In one in vitro study, MLT increased the proliferation of myeloma cells (Persengiev & Kyurkchiev, 1993).

## Mucositis

Mucositis is a common side effect of radiation therapy especially seen during treatment for head and neck malignancies and/or with concurrent chemotherapies that cause mucositis. The development of mucositis may lead to dose limitation, pain, greater chance of infection, and dysphagia, causing difficulty ingesting food and fluid, thus affecting nutrition and hydration status and leading to possible weight loss (Keefe et al., 2007; Worthington, 2011).

**DEGLYCYRRHIZINATED LICORICE**

While studies on Glycyrrhiza (licorice) and its use during RT in particular are limited, it is worth noting for its mucosal healing properties and to stimulate research interest in this potentially valuable therapeutic agent. Deglycyrrhizinated licorice (DGL) has the glycyrrhetinic acid component that is known to possess hypertensive properties removed, resulting in a compound that has only a few rare side effects and is generally recognized as safe by the US Food and Drug Administration (Messier, Epifano, Genovese, & Grenier, 2012). Deglycyrrhizinated licorice is available in wafer form, is easily dissolved, and is cost-effective. This treatment has been shown to be clinically beneficial yet does require frequent dosing for effectiveness.

Clinical trials have shown DGL to be as effective in healing gastric and duodenal ulcers as carbenoxolone, cimetidine, and ranitidine (Mills & Bone, 2000). A preliminary study by Das, Das, Guati, and Singh (1989) reported on the use of a mouthwash containing DGL extract for 2 weeks. The treatment provided pain relief and accelerated the healing of aphthous ulcers. In a randomized, double-blind clinical trial, subjects with recurrent aphthous ulcers were assigned to receive either a patch with extract of glycyrrhiza root, a placebo patch, or no treatment at the onset of a lesion. Ulcer size (* p* < .05) and pain (* p* < .01) improved with the extract when compared to the placebo and no-treatment groups (Martin, Sherman, van der Ven, & Burgess, 2008).

The authors were only able to find one study pertaining to RT and licorice. A total of 75 patients receiving radiotherapy to the head and neck were divided into 4 groups, each receiving local application of various substances to the oral cavity just prior to their treatment. For 7 weeks, group A patients applied licorice powder and honey locally each day and consumed 10 mL of a licorice preparation twice a day; group B applied licorice powder and honey locally; group C applied only honey locally; and group D (control) received standard medical treatment for mucositis. Group A had the greatest reduction in radiation- and chemotherapy-induced mucositis (* p* < .001) compared to the control group (Das, 2011). Perhaps the beneficial effect was also due to the honey, as seen with the trials mentioned on page 226. This study does not mention blinding for the evaluation of mucositis, but the Radiation Therapy Oncology Group/European Organisation for Research and Treatment of Cancer (RTOG/EORTC) grading systems were used. The crude substances for the three groups were made in-house and may not be reproducible in future studies.

The conclusions drawn are not complete; for example, it is mentioned that with licorice use there were no interruptions in treatment and food intake was not severely affected, but there were no data showing these outcomes in the other three arms of the study for comparison. While there are flaws in the methods, to date this was the only study of this potentially important botanical for the alleviation of radiation-induced mucositis found in the published literature. More studies are needed for further research on this botanical.

**GLUTAMINE**

Glutamine is a nonessential amino acid. It is the primary fuel for enterocytes in normal and stressed states (Savarese, Savy, Vahdat, Wischmeyer, & Corey, 2003). A Cochrane review on preventing oral mucositis showed no statistically significant benefit of using oral glutamine and weak evidence for IV glutamine in preventing severe mucositis (Worthington et al., 2011). In 2008, the American Cancer Society released guidelines for the prevention and treatment of mucositis and recommended against the use of systemic glutamine on the basis that multiple trials had produced conflicting results (Keefe et al., 2007). One trial referenced in these guidelines resulted in more severe mucositis and a non–statistically significant increase in mortality at 2 years in the glutamine-treated group. This study involved the use of IV alanyl-glutamine dipeptide and was performed on patients undergoing bone marrow transplantation.

Recently, however, a systematic review by Gibson et al. (2013) analyzed the available literature and defined evidence-based clinical practice guidelines for the use of agents for the prevention and treatment of mucositis. According to the authors, perhaps one of the most important findings from the review was the change in guideline for the use of systemic glutamine. New literature demonstrates that glutamine may, in fact, be effective and without severe toxicity. Due to conflicting evidence at this time, the guideline on glutamine has been changed from "not recommended" to "no guideline possible." New research in this area is eagerly anticipated.

Smaller trials not included in the review articles suggest a benefit for using glutamine during radiation (Savarese et al., 2003). Contact with the mucous membranes as swish and swallow, as well as duration of treatment, is what provides protective effect against mucositis/stomatitis (Noé, 2009; Savarese et al., 2003). However, IV glutamine also seems to produce this protective effect in a small trial (Worthington et al., 2011). Conclusions from phase I and II pilot studies regarding dosage guidelines show that 20 to 30 g daily of glutamine in divided doses is effective (Noé, 2009). It is most effective when given at the start of radiation until 2 weeks after completion. Caution should be used with hepatic encephalopathy or hyperammonemia, as intestinal glutamine catabolism produces about 50% of the ammonia released into the portal vein.

**HONEY**

A recent systematic review and meta-analysis showed an 80% relative risk reduction in radiation-induced oral mucositis in honey-treated patients compared with control patients (Song, Twumasi-Ankrah, & Salcido, 2012). The meta-analysis included three studies in which patients with head and neck cancers receiving RT were evaluated for radiation-induced mucositis using the RTOG (Table 2) and WHO criteria (Table 3). In total, 60 patients (20 patients per study) applied honey to the inside of their mouths before, directly after, and several hours after RT. Another 60 patients were part of the control groups that did not actively receive treatment. The risk of developing mucositis in the honey-treatment group was 80% lower than in the control group (relative risk [RR], 0.19; 95% confidence interval [CI] = 0.098–0.371). Due to risk of bias in these studies, the authors recommended approaching this therapy with caution. A Cochrane review shows benefit for the prevention of mucositis (RR, 0.70, 85% CI = 0.56–0.88;* p* = .002) and also advises caution (Worthington et al., 2011). Further studies determining specific clinical recommendations are needed, but one study used a 20-mL honey swish and swallow before, directly after, and again 6 hours after RT (Biswal, Zakaria, & Ahmad, 2003).

**Table 2:**
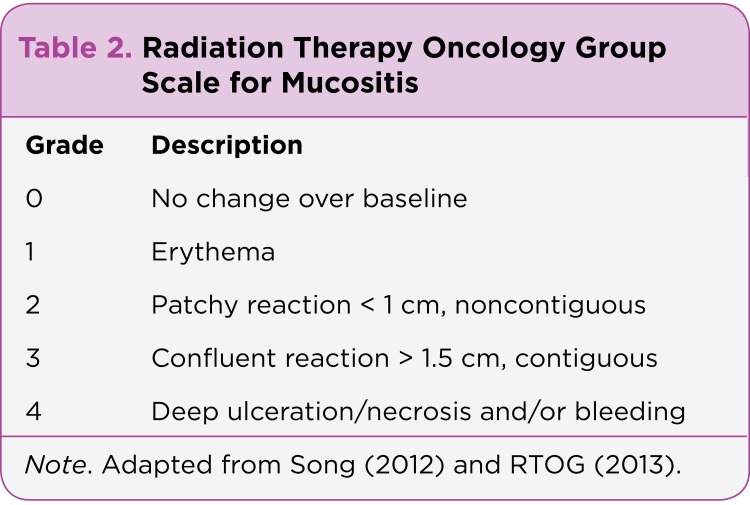
Table 2. Radiation Therapy Oncology Group Scale for Mucositis

**Table 3:**
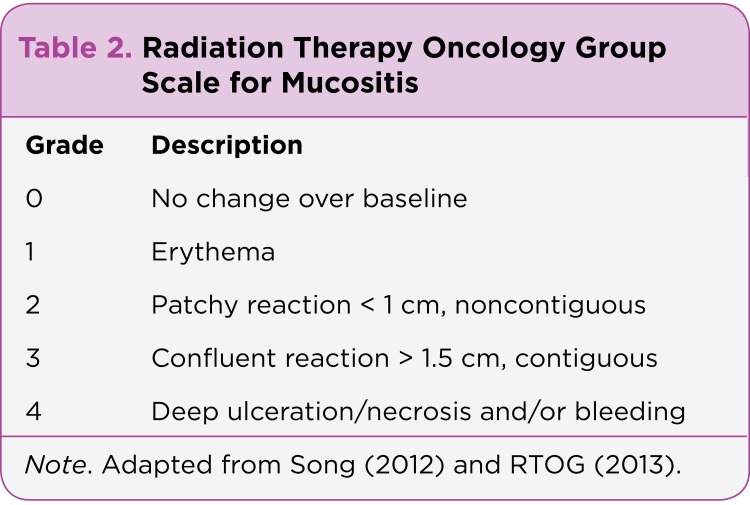
Table 3. World Health Organization Scale for Oral Mucositis

Radiation therapy puts patients at increased risk of forming dental caries. Santos-Silva, Rosa, Eduardo, Dias, & Brandao (2011) have commented regarding the concern that the intake of honey may perpetuate this risk. Perhaps prophylactic measures to prevent and offset the risk of caries due to treatment with honey would encourage the conservative practitioner to use this promising therapy.

The topical application of natural honey is a simple and cost-effective treatment for radiation mucositis that warrants further multicenter randomized trials. Future research in this area should address issues of bias found in many of the preliminary honey studies. Blinding bias, for example, may be difficult to overcome due to the distinct taste and texture of honey and the difficulty of creating a honey-like substitute for a control group.

## Skin Toxicity

Skin changes can occur in up to 95% of patients undergoing RT, which may be a dose-limiting side effect for some individuals (McQuestion, 2011). Patients with a higher risk of skin reaction include those receiving treatment to the breast, perineum, axilla, and face, as well as to areas of disrupted skin integrity such as from surgery, burns, or other lesions. A number of recommendations made for skin applications across many treatment centers are based on limited published evidence or anecdotal evidence, although these recommendations would not be expected to cause harm (Kumar, Juresic, Barton, & Shafiq, 2010; McQuestion, 2011). Studies that show significant benefit of topical applications are limited.

**ALOE VERA**

Although aloe vera is widely recommended, there is insufficient evidence to support its use in treatment for RT skin side effects (Kumar et al., 2010; McQuestion, 2011). A survey of multiple RT departments showed that 60% of the participants in the survey recommended aloe vera based on anecdotal evidence (Kumar et al., 2010). Reviews evaluating the effectiveness of aloe vera in reducing radiation skin reactions showed no benefit of aloe vera gel over the control arm (Kumar et al., 2010; McQuestion, 2011). In one study by Heggie et al. (2002), dry desquamation increased in the aloe vera group compared to the control group (70% vs. 41%). This phase III study evaluating the efficacy of topical application of aloe vera gel vs. aqueous cream on irradiated breast tissue found that dry desquamation was significantly lower in the control grou* p* <italic>(*p* =.001) as well. The aloe vera arm showed a significantly greater probability of grade 2 or greater pain *(p* =.03). The use of aloe vera in the treatment of radiodermatitis is not recommended, as evidence to suggest benefit is lacking.

**CALENDULA**

A review and meta-analysis of several agents used to manage skin toxicity during RT showed a statistically significant benefit of using calendula (Kumar et al., 2010). One study of patients receiving RT for breast cancer showed a statistically significant reduction (* p* < .001) in the occurrence of grade 2 or higher toxicity using the RTOG grading system (Table 4) compared with trolamine, a nonsteroidal topical agent that enhances skin healing by immunomodulation (Pommier et al., 2004). Further, patients experienced reduced pain related to skin reactions (* p* < .03) and reduced incidence of treatment interruption. The product used in this study was an accessible, cost-effective, over-the-counter homeopathic calendula ointment. Thirty percent of the patients in this study believed application of the cream to be difficult. Topical application began at the onset of RT and was applied twice daily or more through the completion of RT. This study demonstrates that there is sufficient evidence for the use of calendula cream as a preventive agent.

**Table 4:**
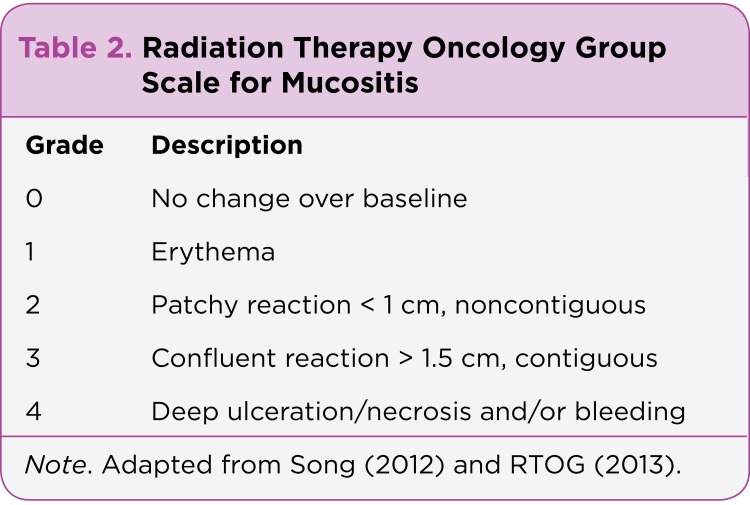
Table 4. Radiation Therapy Oncology Group Acute Skin Toxicity Grades

With this type of preparation, APs should advise their patients to apply it only after radiation has been administered, as the skin should be dry and clean prior to RT, to prevent any interference from oil-based applications. While clinical studies were not found related to managing proctitis with calendula ointment, the authors have personally seen beneficial results with patients who use this type of preparation during RT to the pelvic region.

## Xerostomia

Xerostomia, or dryness in the mouth due to lack of normal salivary secretion, is a serious side effect of radiation that can be acute or late (Bruce, 2004). Acute effects may present during treatment or up to 3 months after treatment and is usually self-limiting. Late effects may appear more than 3 months after RT ends and may be permanent. Pilocarpine, which is commonly used to treat xerostomia, is a cholinergic parasympathomimetic agent shown to enhance salivary secretion by stimulating muscarinic receptors on the surface of salivary gland cells (Jensen et al., 2010). While it has been extensively researched, pilocarpine offers modest effectiveness and an array of adverse cholinergic effects such as sweating, nausea, rhinitis, and chills, which limit its use (Lin & Chen, 2012). Alternatives with fewer side effects are necessary and recommended.

**ACUPUNCTURE**

The oral care study group of the MASCC/ISOO produced evidence-based management recommendations for treating and/or preventing xerostomia (Jensen et al., 2010). This panel suggests the use of acupuncture to stimulate salivary flow and relieve xerostomia with a rating of level II evidence and grade C recommendation. It is generally accepted that acupuncture is safe and not associated with serious side effects. Although investigations to determine the most reliable acupuncture technique are in progress, the studies discussed below and summarized in Table 1 have all shown promising results.

While studies also vary on the number and timing of acupuncture visits, it seems that patients do better with frequent follow-up (Bruce, 2004; Jensen et al., 2010; Lin & Chen, 2012). Many studies have shown the duration of the beneficial effects of acupuncture to be from months to years on follow-up. One study of patients who had completed RT and were refractory to pilocarpine found responders requiring twice the number of visits as nonresponders, and suggested a regimen of three to four weekly treatments followed by monthly sessions (Johnstone, Niemtzow, & Riffenburgh, 2002). One small trial found that preventive acupuncture prior to and during RT was beneficial for minimizing severity of xerostomia-related symptoms (Braga, Lemos, Alves, & Migliari, 2011). In this study, acupuncture was administered twice weekly for 16 to 20 sessions.

## Discussion

Given the widespread interest in CAM strategies during cancer treatment, communication regarding CAM is important for both the patient and the provider. One survey showed that physicians feel uncomfortable discussing questions about CAM treatments due to their lack of knowledge about the subject (Ge et al., 2012). Many cancer centers are now employing practitioners who have the appropriate training to safely advise patients on this topic. For a summary of evidence supporting CAM use in RT, refer to Table 1.

Among the therapies discussed, reduced incidence and severity of diarrhea have been observed in patients taking psyllium or probiotics. There is weak evidence that glutamine prevents diarrhea with RT alone, and may be considered in cases of concurrent chemoradiation to ameliorate diarrhea and other chemotherapy-related side effects. Melatonin has been shown to improve survival at 1 year and increased quality of life in patients undergoing chemotherapy and RT. Melatonin was also shown to improve fatigue and may be helpful for treating insomnia. Further, the SERM and SEEM properties of MLT may be of added benefit for patients with breast cancer. Research shows exercise is safe during and after RT and can improve fatigue. For mucositis, one might consider using honey, with preventive care guidelines for dental caries, whereas glutamine does not appear at this time to provide any benefit for radiation-induced mucositis alone. Patients undergoing concurrent chemoradiation therapies might benefit from the use of glutamine to treat mucositis and other side effects of chemotherapy.

Deglycyrrhizinated licorice has been used successfully in treating ulcerations of the GI tract, but there is not enough research to substantiate its use for radiation-induced mucositis. It is, however, considered a safe product; future studies on this promising therapy are needed. Skin toxicity can be managed with calendula cream, but aloe vera gel is not recommended, as there has been no observed therapeutic benefit. Xerostomia is a difficult radiation-induced side effect to treat, and acupuncture has shown benefit for treatment of this condition.

Therapies must be considered on a case-by-case basis. Taking into consideration a palliative vs. curative intent might change the willingness of the practitioner to use a therapy with the potential to interfere with long-term survival if it alleviates the negative side effects of radiation and improves quality of life.

## Conclusions

As mentioned in the introduction, CAM encompasses a wide range of modalities. Certain modalities—such as spiritual healing and prayer, for example—would not be expected to negatively interfere with efficacy of treatment. In the study by Rausch et al. (2011), spiritual healing/prayer was most reported by patients, followed by multivitamins. While it is important to focus the research on supplements and other nutritional substances that may have the potential to alter RT outcomes, it is also important to examine other CAM therapies patients are utilizing and consider a more comprehensive cancer treatment program that makes such strategies more available for patients’ benefit.

A team approach to the management of each patient is beneficial for all parties involved. Many cancer centers are currently incorporating this model of care, blending conventional treatment strategies with CAM modalities. Due to the large percentage of patients seeking CAM, it seems appropriate for cancer centers to consider offering these services to meet their patients’ needs.

As with much CAM research, more rigorous studies are needed to confirm the results of the studies discussed in this review as well as to definitively establish safe and effective dosage and administration.

## Acknowledgments

The authors would like to thank the talented group who helped with this article: Kate Barton-Miyagi, ND, Christina Amicone, ND, Margaret Beeson, ND, and Adeola Mead, ND.
